# Homeless Outreach Psychiatric Engagement for Lisboa (HOPE 4 Lisboa): One year of marontology, and one John Doe living with Diogenes syndrome

**DOI:** 10.1177/00207640231179322

**Published:** 2023-06-22

**Authors:** João Gama Marques, Daniela Chesi, Raquel Oliveira Coelho, Inês Castanheira Costa, Celso Santos Antão, Carlos Alberto Pedro, Paulo Silva Santos, José Xavier Diogo

**Affiliations:** 1 Consulta de Esquizofrenia Resistente, Hospital Júlio de Matos, Centro Hospitalar Psiquiátrico de Lisboa, Lisboa, Portugal; 2 Clínica Universitária de Psiquiatria e Psicologia Médica, Faculdade de Medicina, Universidade de Lisboa, Lisboa, Portugal; 3 Department of Neuropsychology and Psychopharmacology, Maastricht University, Maastricht, Limburg, Netherlands; 4 Equipa de Missão do Plano Municipal para a Pessoa em Situação de Sem Abrigo, Câmara Municipal de Lisboa, Lisboa, Portugal; 5 Departamento de Saúde Pública, Administração Regional de Saúde de Lisboa e Vale do Tejo, Lisboa, Portugal

**Keywords:** Homeless, outreach, psychiatry, engagement, John Doe, Diogenes

## Abstract

**Background:**

In Europe, psychiatric disorders seem to affect up to 50% of the homeless. In Portugal there were, at a certain time, circa 3,396 homeless people, half living in the capital city, Lisboa.

**Aims:**

The Homeless Outreach Psychiatric Engagement for Lisboa (HOPE 4 Lisboa) was created, in January 1st 2022, as a collaboration including staff from the local state asylum, medical school and town hall in Lisboa, Portugal, in order to provide better treatment for the super difficult cases of psychiatric patients living homeless in Lisboa.

**Method:**

During 2022, the HOPE 4 team made night rounds, every 15 days on Tuesday’s night (20:30 to 23:30) trying to reach, at least, one dozen of homeless psychiatric patients, previously identified.

**Results:**

The HOPE 4 Lisboa interviewed 101 patients (53.4%) out of the 189 programed visits. From this group, 72 (72%) had already a previous psychiatric diagnosis. From those 101 patients, reports for 47 (47%) were sent for an eventual compulsory psychiatric assessment. From those 47 only 21 patients (21%) were admitted in the psychiatry ward. Finally we discuss the most super difficult patient we found, as a small case report: a John Doe living in complete Diogenes syndrome.

**Conclusions:**

there are still a few psychiatrists interested in treating homeless people completely or partially out of the classic mental health care systems. Some claim to be doing interstitial psychiatry, others street psychiatry, but we could also call it marontology.

## Introduction

In Europe, psychiatric disorders seem to affect up to 50% of the homeless (Barreto et al., 2019). In Portugal there were, at a certain time, circa 3,396 homeless people, half living in Lisboa , the capital city (Gama Marques & Bento, 2020a). The Homeless Outreach Psychiatric Engagement for Lisboa (HOPE 4 Lisboa) was created, in January 1st 2022, as a collaboration including one psychiatrist from the state asylum, at *Consulta de Esquizofrenia Resistente, Hospital Júlio de Matos, Centro Hospitalar Psiquiátrico de Lisboa (CHPL)* and one of local medical schools, *Faculdade de Medicina da Universidade de Lisboa (FMUL)*, plus various members of the *Equipa de Missão do Plano Municipal para a Pessoa em Situação de Sem Abrigo*, of the town hall at *Câmara Municipal de Lisboa (CML)*. European level supervision of HOPE 4 Lisboa has been provided by senior members at the *Santé Mentale et Exclusion Sociale Europa (SMES Europa).*

## Methods

During 2022, the CHPL psychiatrist led the HOPE 4 team, every 15 days, on Tuesday’s night (20:30 to 23:30), with the collaboration of the CML staff (e.g. psychologist, nurse, social worker, sociologist and/or anthropologist). On a nine seat minibus van, they tried to reach at least one dozen of homeless psychiatric patients, previously identified by the *CML*, keeping the tradition started more than 20 years ago by Dr. António Bento (Neves Horácio et al., 2023). Besides the general results we will also discuss a case vignette of one of our most difficult cases: an elderly psychotic John Doe living with Diogenes syndrome.

## Results

### General results

The HOPE 4 Lisboa did 19 outreach visits, looking for 189 individuals (151 males). One fourth (24.3%) were living roofless in Arroios, the Lisboa’s equivalent to New York’s Bowery, or the Los Angeles’ Skid Row. Besides Portugal (74%) the homeless list included people from 17 different nationalities: the Portuguese ex-colonies such as Brazil, Angola, Cape Verde, Guinea Bissau, Mozambique, or Saint Thomas and Prince (16%) and other countries like Bulgaria, The Gambia, India, Netherlands, Nigeria, Russia, Saudi Arabia, Switzerland, Ukraine and United States of America (10%).

**Figure 1.**
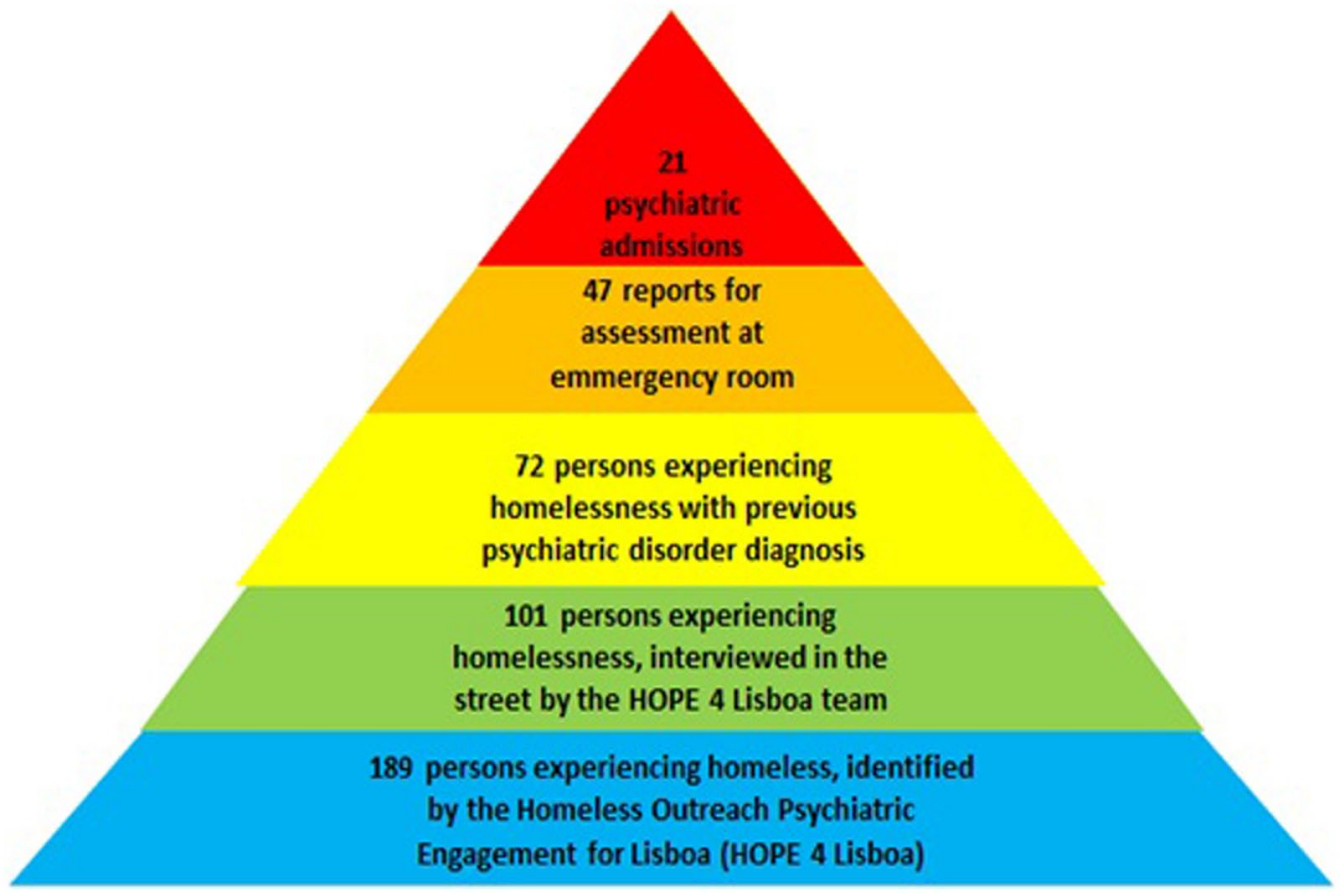
Homeless outreach psychiatric engagement for Lisboa (HOPE 4 Lisboa).

The HOPE 4 Lisboa interviewed 101 patients (53.4%). From this group, 72 (72%) had already a previous psychiatric diagnosis, at the *CHPL* electronic clinical records. From those 101 patients, reports for 47 (47%) were sent asking the public health authorities, for a compulsory psychiatric assessment. Therefore, they were taken by the police, to the general hospital emergency room, for observation followed by admission or discharge, decided by an independent team of psychiatrists. From those 47 only 21 patients (21%) were admitted in the psychiatry ward (Figure 1).

The most prevalent diagnoses, according to the World Health Organization’s International Classification of Diseases (WHO ICD-10), were psychosis non other specified (F29) (55.6%), followed by alcohol disorders (F10) (21.2%), and organic psychoses (F06) (13.2%). Together, psychosis non other specified (F29) and organic psychosis due to brain damage (F06) accounted for more than two thirds of the sample (68.8%), in line with our previous works (Monteiro Fernandes et al., 2022). Against our expectation (Spranger Forte et al., 2023), at this point of our work, we were not able to diagnose a single case of schizophrenia neither schizoaffective disorder among the homeless population we visited.

### Case report

Now we present the most difficult patient we visited in the first year of our HOPE 4 Lisboa project. During one of our team cold winter night raids, we were called to visit someone sleeping rough, in a very bad shape, under the bridge, near a busy train station, here in Lisboa, Portugal, Europe.

**Figure 2.**
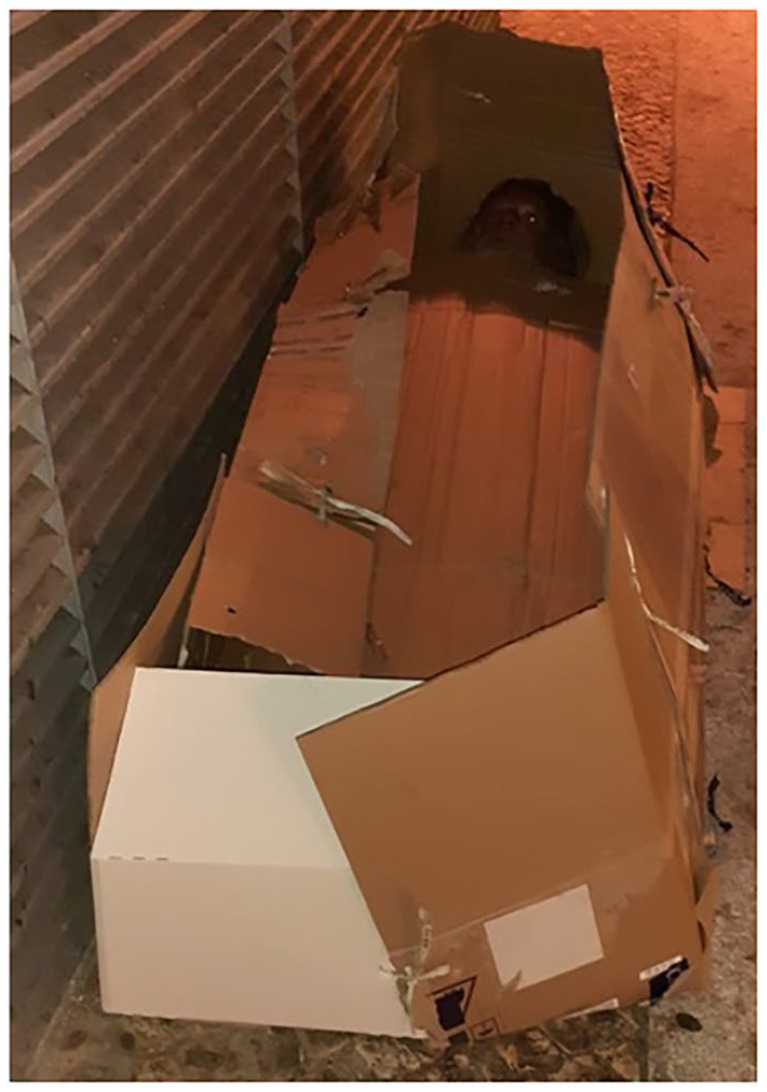
Homeless man, with Diogenes and John Doe syndromes, sleeping in an improvised hand-made cardboard sarcophagus.

At arrival, we were astonished to find an improvised hand-made cardboard sarcophagus (Figure 2). Inside, there was an elderly man in cachexia. His skin and beard were covered by dirt, emanating a strong body odor, a pungent stench, in complete squalor (Diogenes syndrome). He got outside of his sarcophagus, and we realized he was only using one piece of underwear. Dysphoric, irritable, menaced and insulted us in full blown coprolalia, refusing to identify himself (John Doe syndrome). Verbalizing grandiose and persecutory delusions, he claimed to be god and refused any need for help (psychosis non other specified). A complete interview was not possible, unfortunately, for reasons of staff security.

We made a report asking, the public health authorities, for an involuntary, compulsory psychiatric admission, but there was no feedback at all. In the following monthly visits we found no patient, just the empty hand-made cardboard sarcophagus, under the bridge, exactly in the same spot. Like a forgotten nest, an abandoned cocoon. More than 1 year later, there is still no news regarding this patient.

## Discussion

One big, obvious, problem when working with homeless people is that we cannot do classic community psychiatry visits or the usual outpatients appointments, when a person has neither a home, a phone number or even an electronic address. This shows one methodological issue when dealing with those patients, especially since they often need the most complex care. Besides dealing with psychiatric disorders and heavy clinical comorbidity (Carnot & Gama Marques, 2018), the HOPE 4 Lisboa team finds a lot of social and administrative problems such as inadequate shelter (Gama Marques, 2022a), or even the anonymity of patients (Gama Marques, 2021). And the anonymity of patients brings us another wicked problem.

On the last 30 years some authors have been claiming that the medicalization of homelessness has been used to divert attention from socioeconomic problems, and to justify the removal of homeless people from public spaces (Mathieu, 1993). Other authors have been arguing that the closing of public psychiatric hospitals (deinstitutionalization) caused no homelessness at all, and that the psychiatric abandonment of patients, who once were housed in large institutions, should be regarded as a myth or a sacred cultural tale (Mossman, 1997). Even psychiatrists working with persons experiencing homelessness started questioning. Shall we respect their right to dwell neglected in the streets? Shall we respect their inability to comprehend and accept housing, refusing medical care, and human dignity? Shall we respect their quest for anonymity? (Melamed et al., 2000).

This kind of opinions certainly had some impact in the Western, Educated, Industrialized, Rich, and Democratic (WEIRD) societies (Henrich et al., 2010). Fortunately, some psychiatrists never stopped worrying and working with the homeless, the so called fourth world (Raps & Kemelman, 1994). And today the epidemiological definition for fourth world still includes those individuals: ‘The environmental and socioeconomic situation of decayed urban neighborhoods in affluent nations, resembling the conditions encountered in the poorest developing countries. It includes homeless people, who are among an underclass (often disenfranchised) found in urban communities in rich countries’. (Porta, 2016).

## Conclusion

Professionals working with homeless people deserve more recognition and support from the government side, as well as from civilian society. They need more support to provide appropriate treatment and housing. Although unimaginable difficulties, there are still a few psychiatrists interested in treating homeless people, completely or partially, out of the classic mental health care systems. Some claim to be doing street psychiatry (Lo et al., 2022), others call it interstitial psychiatry, as teached by Prof. Glenn J. Treisman at the Johns Hopkins University’s Department of Psychiatry and Behavioral Sciences (Redgrave & Ruble, 2021). We could also call it marontology, a still to be created medical specialty, deriving from *marontos* (the Greek word for unwanted) (Gama Marques, 2021), dedicated to the most super difficult patients (Gama Marques, 2019, 2022b; Gama Marques & Bento, 2020b) we have encounter in this journey.
